# The Protective Effect of a Human Umbilical Cord Mesenchymal Stem Cell Supernatant on UVB-Induced Skin Photodamage

**DOI:** 10.3390/cells13020156

**Published:** 2024-01-15

**Authors:** Lin Cheng, Jiaqi Liu, Qi Wang, Huozhen Hu, Liming Zhou

**Affiliations:** 1 Department of Pharmacology, West China School of Basic Medical Sciences and Forensic Medicine, Sichuan University, Chengdu 610041, China; chenglin2022@stu.scu.edu.cn (L.C.); wangqi@stu.scu.edu.cn (Q.W.);; 2 Key Laboratory of Environmental Pollution and Integrative Omics, Education Department of Guangxi Zhuang Autonomous Region, Guilin Medical University, Guilin 541001, China; liujiaqi@glmc.edu.cn

**Keywords:** human umbilical cord mesenchymal stem cell supernatant, UVB, skin photodamage

## Abstract

The skin is constantly exposed to a range of environmental stressors, including ultraviolet (UV) radiation, which can cause damage to the skin. Repairing UV-damaged skin has been a major focus of research in recent years. The therapeutic potential of human umbilical cord mesenchymal stem cells (HUCMSCs) exhibits anti-photoaging properties. In this study, we developed a strategy for concentrating an HUCMSC supernatant, and examined the protective effects of CHS on UVB exposure in vitro and in vivo. Our results demonstrate that CHS repairs UVB exposure by promoting cell viability and migration and reducing senescent and apoptosis cells. We further found that the photoprotective effect of CHS is due to autophagy activation. Moreover, CHS reduces wrinkles and senescent cells, increases collagen expression, and improves immune function in UVB exposure-induced skin damage. In summary, our study provides a new approach for repairing cell damage, and suggests that CHS might be a potential candidate for preventing UVB-induced skin photodamage.

## 1. Introduction

Long-term exposure to ultraviolet (UV) irradiation leads to several potentially harmful effects on human skin, including sunburn, skin aging, and skin cancers [1]. UV radiation is typically divided into three wavelength bands: UVA (320–400 nm), UVB (280–320 nm), and UVC (100–280 nm) [2]. Notably, UVB irradiation is the most cytotoxic and mutagenic stress-inducing electro-magnetic wave, and results in pathological alterations such as sunburn, erythema, and edema, ultimately contributing to skin photoaging [3,4]. The harmful effects of UVB irradiation lead to DNA damage, and thus directly affect cells’ proliferation and healing ability, therefore increasing the risk of exposed individuals to develop skin cancer [5,6]. To repair UVB irradiation-induced skin damage, autophagy, a catabolic process that clears damaged proteins and organelles, was activated, thereby reducing cell death [7]. UVB irradiation of skin dermal fibroblasts can induce the formation of autophagosomes to inhibit cell apoptosis and senescence, serving as a protective mechanism against cell death [8,9]. Thus, the potential significance of modulating autophagy levels is an intervention strategy to mitigate the adverse effects of UVB irradiation.

In modern skincare, the application of skin damage-suppressing and self-renewing reagents would be a promising avenue to protect against UVB irradiation-induced skin photoaging and DNA damage [10]. Mesenchymal stem cells (MSCs) are a type of adult stem cell that can self-renew and differentiate into various cell and tissue types, which can be applied to damaged and aged skin based on its strong healing capacities [11,12]. The International Society for Cellular Therapy (ISCT) has suggested at least three conditions that can characterize MSCs: MSCs must adhere to a plastic culture vessel and grow; MSCs should have CD73, CD90, and CD105 as cell surface antigens, and CD11b, CD14, CD19, CD34, CD45, CD79α, and HLA-DR antigens should not exist on MSCs; and MSCs must be able to differentiate into osteoblasts, adipocytes, and chondrocytes in vitro [12,13]. Generally, MSCs have been isolated from numerous tissues, including adipose tissue, skin, dental tissue, umbilical cord (UC) tissue, and umbilical cord blood (UCB) [14]. Human UC-derived MSCs (HUCMSCs) are the most promising and widely used source because of their strong migratory activity, paracrine function, and low immunogenicity [15]. HUCMSC-derived soluble factors have demonstrated therapeutic potential, with anti-photodamage effects to prevent skin aging [16,17]. Based on previous research findings, the utilization of MSC-derived molecules to repair damaged and aging skin exhibits substantial potential within the realm of therapeutic interventions.

In this study, we utilized a concentrated supernatant of HUCMSCs (CHS) to investigate its protective effects on UVB irradiation-induced skin damage and aging. Experimental validation in vitro, including cell viability, cell cycle, cell migration, and cell senescence, was performed to determine the therapeutic effect of CHS on skin photoprotection. We also discussed the photoprotective effects of CHS that correlate with the activation of autophagy. Furthermore, our results revealed that CHS provides significant protection against UVB-induced skin damage in mice. In this study, we aimed to find out the protective effects of CHS application, which could help further research into the mechanisms underlying UVB irradiation-induced skin photodamage therapy. 

## 2. Methods and Materials

### 2.1. Isolation and Identification of HUCMSCs

HUCMSCs were obtained from the Chuanbei Medical College Ethics Committee. The umbilical cord (UC) was washed with phosphate-buffered saline (PBS), and we then removed the peripheral fascia, arteries, and vein. The UC was then chopped into smaller pieces and cultured in α-MEM media (Gbico, Billings, MT, USA) supplemented with 10% fetal bovine serum (FBS) (PAN, Munich, Germany) in a 5% CO_2_ humidified incubator at 37 °C. The surface markers of the HUCMSCs were evaluated using flow cytometry with CD73-PE, CD90-FITC, CD105-PE, HLA-DR-FITC, CD19-PE, and CD11b-FITC monoclonal antibodies (Beckman Coulter, Brea, CA, USA). And the cells with specific markers, namely CD105^+^, CD73^+^, CD90^+^, CD11b^−^, CD19^−^, and HLA-DR^−^, were sorted and collected. The differentiation potential of the HUCMSCs was assessed using osteogenic, lipogenic, and chondrogenic induction kits (iCell, Shanghai, China). Accessing the quality of the metaphases of HUCMSCs requires a classical G-banding analysis, which was used to detect chromosomal abnormalities on the HUCMSCs, as previously reported [18]. 

### 2.2. CHS Collection and Treatment

The HUCMSCs were cultured in α-MEM media supplemented with 5% human platelet lysate (Compass Biomedical, Hopkinton, MA, USA) to obtain a supernatant. The cell supernatant was concentrated using a dialysis bag and then inserted into a beaker filled with polyethylene glycol (PEG) 8000 at 4 °C. The concentrated HUCMSC supernatant (CHS) was shrunk to 1/45 of its original volume. The CHS was filtered using a 0.22 μm filter tip, collected in sterile centrifuge tubes, and frozen at 4 °C for short-term storage or −20 °C for long-term storage. The protein content of the CHS was determined via a BCA assay (Thermo Fisher, Pleasanton, CA, USA). The CHS was diluted to different concentration for cell experiments, including 0%, 2.5%, 5%, 10%, and 20%.

### 2.3. UVB-Damage Cell Model Establishment and Cell Viability Measurement

The immortalized human keratinocyte cell line (HaCaT) was cultured in Dulbecco’s modified Eagle’s medium (DMEM) (Cytiva, Amersham, UK) supplemented with 10% FBS (PAN, Munich, Germany), 100 μg/mL of penicillin, and 100 μg/mL of streptomycin at 37 °C in a humidified atmosphere of 5% CO_2_/95% air. For UVB irradiation, equipment with a power of 1282 μW/cm^2^ (Sigma, Burlington, MA, USA) was used. UVB irradiation groups with various doses, including 0, 50, 100, 150, 200, 250, and 300 mJ/cm^2^, were set to assess cell viability. HaCaT cells were seeded in 96-well plates (8 × 10^3^ cells/well) for 24 h, and treated with CHS after UVB irradiation. Cell viability was measured after 24 h and 48 h, and determined via a CCK8 assay (APExBIO, Houston, TX, USA). A total of 10 μL of the CCK-8 reagent was added to each well and then cultured for 2 h. The absorbance was measured at 450 nm using a microplate reader (Bio-Rad, Hercules, CA, USA), and the data were used to calculate cell viability. Each sample had 4 replications.

### 2.4. Wound-Healing Measurement

HaCaT cells were seeded at a density of 3 × 10^5^ cells in 6-well plates overnight. The monolayer was scratched with a 10 μL pipette tip to create a wound. Subsequently, the cells were exposed to UVB (100 mJ/cm^2^) and then treated with different concentrations of CHS for 0 h, 12 h, and 24 h. The migration of the cells was observed and recorded using a microscope, and the area was quantified with the ImageJ software 1.8.0. 

### 2.5. Cell Cycle Measurement

HaCaT cells were treated with different concentration of CHS for 24 h. The cells were then fixed in 70% ethanol overnight, stained with a propidium iodide (PI)/RNase A solution, and analyzed via flow cytometry (BD FACSCalibur, San Jose, CA, USA) at an excitation wavelength of 488 nm.

### 2.6. SA-β-Gal, AO/EB and Monodansylcadaverine (MDC) Staining

HaCaT cells were exposed to UVB (100 mJ/cm^2^) radiation, then treated with CHS for 24 h. For SA-β-Gal (senescence-associated β-galactosidase) staining, the cells were fixed and incubated with a staining solution containing X-gal at 37 °C for two days. For the AO/EB (acridine orange/ethidium bromide) double-staining assay, the cells were stained with a mixture of AO and EB (5 μg/mL) for 10 min in PBS buffer. The MDC (monodansylcadaverine) staining was measured at an excitation wavelength of 335 nm and an emission wavelength of 512 nm. And the fluorescence images were captured using a fluorescence microscope (Zeiss, Oberkochen, Germany).

### 2.7. Western Blotting

To evaluate the protein expression levels of Beclin 1 and LC3, HaCaT cells were harvested and dissolved in a lysis buffer. The total protein content was determined via a BCA assay (Thermo Fisher, Pleasanton, CA, USA). The samples were separated on a 10% sodium dodecyl sulfate–polyacrylamide gel, and probed with primary antibodies (1:500 dilution; Abcam, USA) at 4 °C overnight. The second antibody was administered at room temperature for one hour with goat anti-rabbit IgG (H + L) (1:10,000 dilution; Abcam, Waltham, MA, USA). And the protein expression level was measured using a chemiluminescence detection kit (Beyotime, Shanghai, China). Each sample had 3 replications.

### 2.8. Animal Study

Experiments were conducted on eight-week-old female Kunming mice purchased from the Chengdu Dashuo Experimental Animal Company (Chengdu, China). The mice were randomly assigned to four groups: control, UVB, UVB + Solvent, and UVB + Solvent + CHS. Each group had six mice. The solvent consisted of a matrix solution containing water, EDTA, collagen powder, hyaluronic acid, butylene glycol, and niacinamide. For the treatment, 1% CHS (*v*/*v*) mixed with the solvent was prepared. To build a UVB-damaged skin model, the mice were exposed to UVB (100 J/cm^2^) for seven weeks (5 days per week; 20 min per day for the first week, 30 min per day for the second week, 40 min per day for the third and fourth weeks, and 60 min per day for the last three weeks). From the second week, 200 μL of the solvent (with or without CHS) was applied to the dorsal skin before UVB radiation. Blood tests were conducted from the inguinal veins in EDTA anticoagulation tubes to identify the number of WBCs (white blood cells) and the LPR (lymphocyte percentage).

### 2.9. Skin Histology Examinations

The samples of mice skin and thymus tissue were fixed in 10% paraformaldehyde for 24 h, embedded in paraffin, and cut into 4 μm sections. The sections were deparaffinized with xylene, hydrated with graded ethanol, and stained with hematoxylin–eosin (HE) and Masson’s trichome using a conventional staining technique. The skin tissue samples were embedded in an OTC encapsulant overnight at −80 °C, then cut into 5 μm thick slices using a frozen sectioning machine (Leica, Wetzlar, Germany), stained with a β-Galactosidase staining solution overnight at 37 °C, and then re-stained with eosin.

## 3. Result

### 3.1. Identification of HUCMSCs and Preparation of CHS

**Figure 1:**
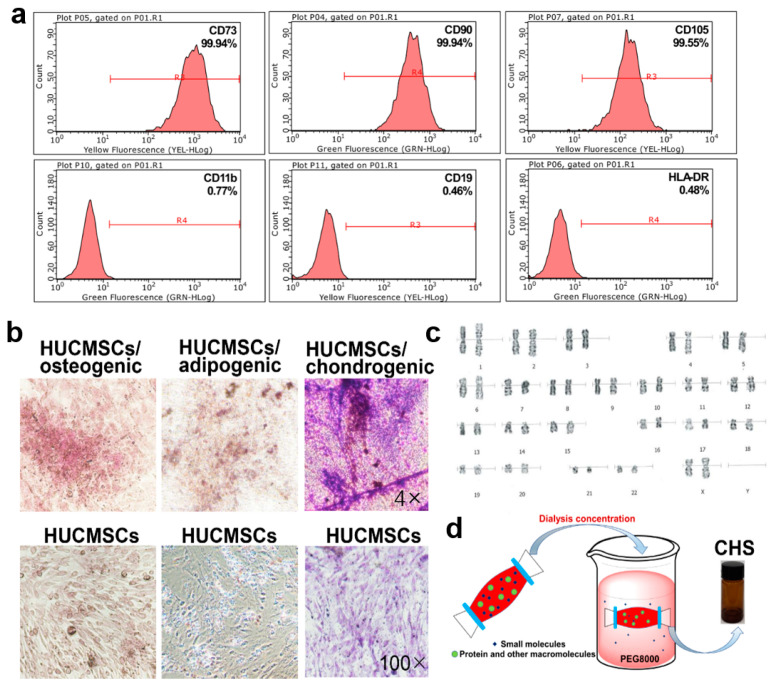
Isolation and identification of HUCMSCs. (**a**) Isolation of HUCMSCs. HUCMSCs were selected based on surface markers (CD73^+^, CD90^+^, CD105^+^, CD11b^−^, CD19^−^, HLA-DR^−^) using flow cytometry. (**b**) Images of tri-lineage differentiation induction in HUCMSCs, including osteogenic, adipogenic, and chondrogenic differentiation (100×). (**c**) Classical G-banding staining of the chromosomal karyotype of P6-generation HUCMSCs. (**d**) Dialysis concentration of HUCMSC supernatant (CHS).

Primary HUCMSCs were isolated from human UC tissue. To confirm the immunophenotype of the HUCMSCs, the HUCMSCs were identified based on their expression of a specific panel of cell surface markers and sorted via flow cytometry (Figure 1a). The positivity rates for CD73, CD90, and CD105 (mesenchymal cell-specific markers) exceeded 99%, while the expression rates of CD11b, CD19, and HLA-DR (mesenchymal cell-negative markers) were less than 1%. The multipotential differentiation properties of the HUCMSCs were confirmed through osteogenesis, adipogenesis, and chondrogenesis experiments in response to appropriate inducement (Figure 1b). We further examined the chromosomal karyotype of the HUCMSCs which could be used for generational cultivation. The results showed that the chromosome structure, number, and shape of the selected cells were similar to typical HUCMSCs (Figure 1c). The supernatant of the HUCMSCs, CHS, was concentrated to remove protein and other macromolecules (Figure 1d). The content of the CHS was 183.18 mg/mL, which can be used in further experiments. 

### 3.2. CHS Improved UVB Irradiation-Induced Decrease in Cell Viability and Migration

**Figure 2:**
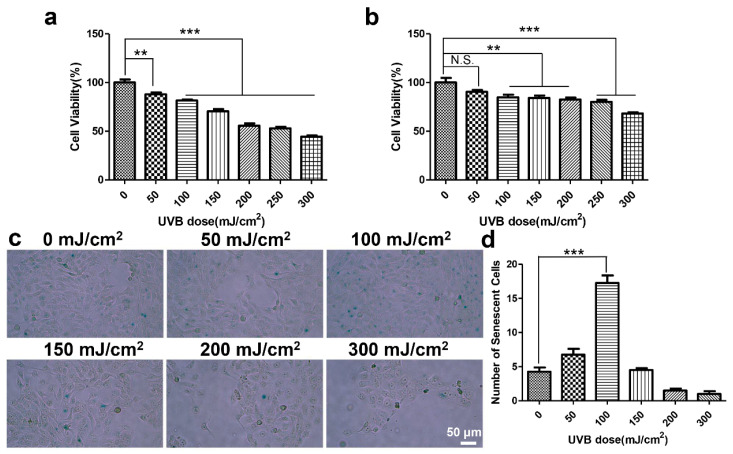
Establishment of UVB-exposure HaCaT cell model. (**a**,**b**) Cell viability of HaCaT cells with different dose levels of UVB exposure after 24 h (**a**) and 48 h (**b**). (**c**) SA-β-Gal staining images of HaCaT cells with different dose levels of UVB exposure after 24 h. (**d**) Statistical data analysis of senescent cells. Scale bar, 50 μm. Results presented as means ± SD of four independent experiments (*n* = 4). ** *p* < 0.01, *** *p* < 0.001, N.S., not significant.

UVB is absorbed by the skin layers and leads to direct DNA damage in epithelial cells. After the HaCaT cells were exposed to UVB irradiation for 24 h and 48 h, the cell viability decreased in a dose-dependent manner (Figure 2a,b). Compared to the control group, we found a significant decrease in cell viability in the 24 h and 48 h groups after having been treated with a UVB dose of 100 mJ/cm^2^ (Figure 2a,b). In addition, we measured the activity of senescence-associated β-galactosidase (SA-β-gal), which is widely used as a major characteristic of senescent cells. The number of senescent cells was significantly increased when treated with a UVB dose of 100 mJ/cm^2^ (Figure 2c,d). These results suggest that exposure to 100 mJ/cm^2^ of UVB irradiation inhibits cell proliferation and promotes cell senescence.

**Figure 3:**
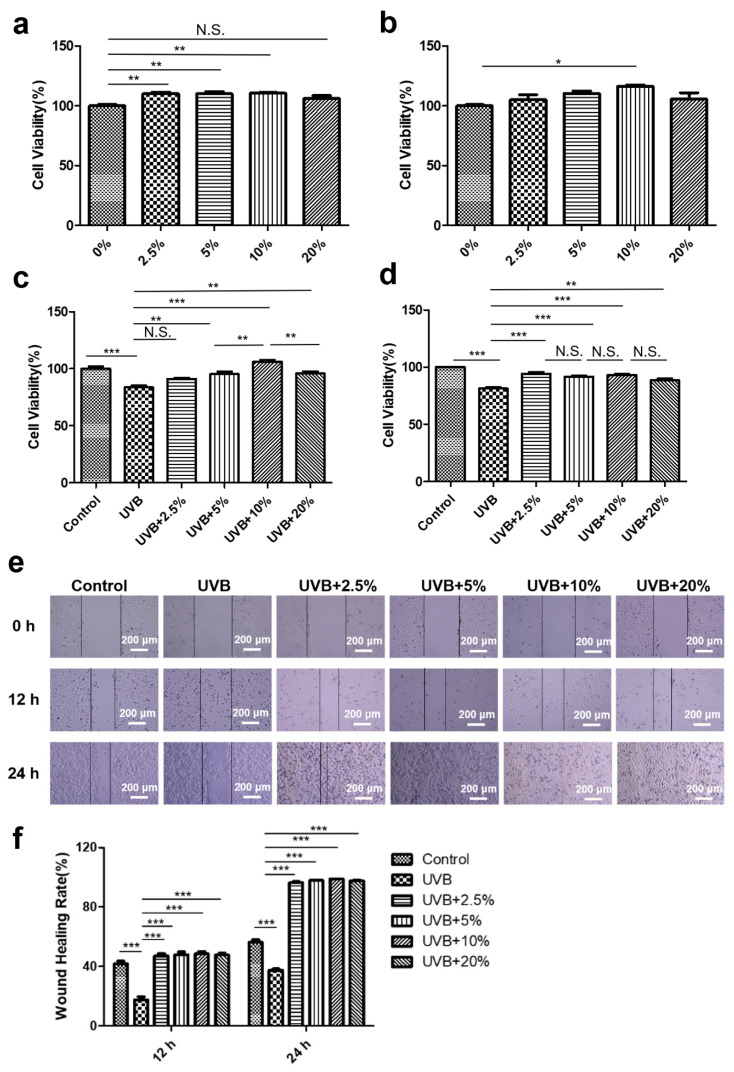
Effect of CHS on viability and migratory ability of HaCaT cells following UVB exposure. (**a**,**b**) Cell viability of HaCaT Cell treated with CHS after 24 h (**a**) and 48 h (**b**). (**c**,**d**) Cell viability of HaCaT cells exposed to UVB radiation with CHS treatment after 24 h (**c**) and 48 h (**d**). (**e**) Wound-healing assay of HaCaT cells exposed to UVB radiation with CHS treatment. (**f**) Scratch-healing rate of HaCaT cells exposed to UVB radiation with CHS treatment. Scale bar, 200 μm. Results presented as means ± SD of four independent experiments (*n* = 4). * *p* < 0.05, ** *p* < 0.01, *** *p* < 0.001, N.S., not significant.

To evaluate the safety of the CHS, HaCaT cells were treated with different concentrations of CHS (0%, 2.5%, 5%, 10%, and 20%) for 24 and 48 h. The CCK8 assay showed that treatment with 2.5%, 5%, and 10% CHS promotes cell growth after 24 h, while being treated with 20% CHS had no effect on cell viability (Figure 3a). The 48 h treatment with 10% CHS had a positive impact on cell viability (Figure 3b). We further examined the effect of CHS on cell viability and cell migration after UVB irradiation. After UVB radiation, the viability of the HaCaT cells was significantly decreased, which could be rescued by treatment with 5%, 10%, and 20% CHS for 24 h (Figure 3c). Similarly, the protective effect of CHS on repairing UVB irradiation-induced cell damage was also observed after 48 h (Figure 3d). The wound-healing assay was used to determine the effect of CHS on the migratory ability of UVB-irradiated HaCaT cells. The wound-healing rates in the CHS treatment groups were significantly higher than those without CHS treatment (Figure 3e,f). Notably, a low concentration of CHS (2.5%) had a protective effect on UVB irradiation-suppressed cell migration. These results indicate that CHS enhances cell survival and migratory potential after UVB irradiation.

### 3.3. CHS Rescues UVB Irradiation-Induced G1 Phase Arrest in Cell Cycle 

**Figure 4:**
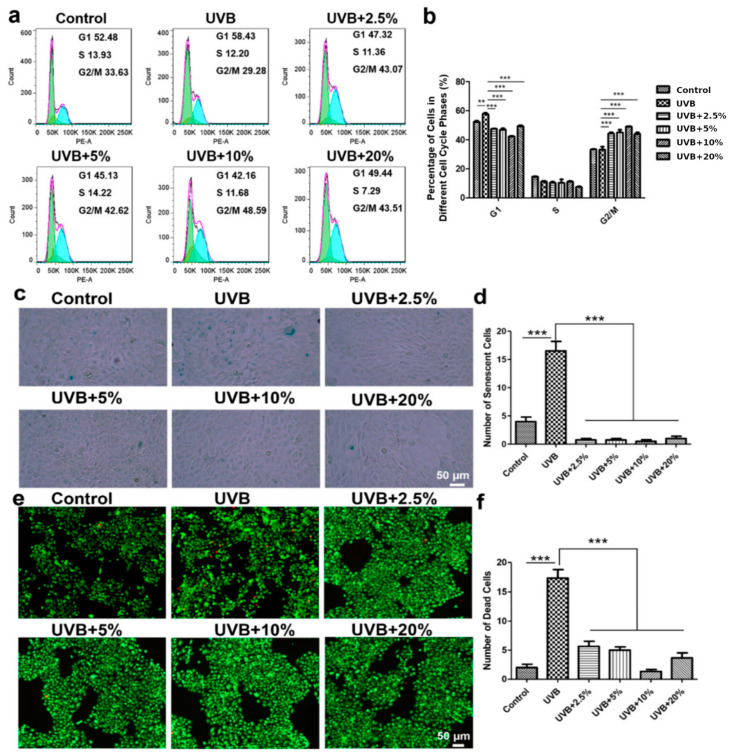
Effects of CHS on cell cycle and cell apoptosis in HaCaT cells following UVB exposure. (**a**) Flow cytometry was used to detect the cell cycle after PI labeling. The vertical coordinates represent effective cell counts. G1, S, and G2/M represent gap 1 phase, synthesis phase, and gap 2/mitotic phase, respectively. Different concentrations (0%, 2.5%, 5%, 10%, and 20%) of CHS were used after UVB-irradiation. (**b**) Percentage of live cells in each of the cell cycle phases of HaCaT cells exposed to UVB radiation with CHS treatment. (**c**) SA-β-Gal staining images of HaCaT cells exposed to UVB radiation with CHS treatment. (**d**) Number of senescent HaCaT cells exposed to UVB radiation with CHS treatment. (**e**) AO/EB staining images of HaCaT cells exposed to UVB radiation with CHS treatment. Green color represents live cells, while red color represents dead cells. (**f**) Number of dead HaCaT cells exposed to UVB radiation with CHS treatment. Scale bar, 50 μm. Results presented as means ± SD of three independent experiments (*n* = 3). ** *p* < 0.01, *** *p* < 0.001.

The UVB-induced decrease in cell proliferation could be associated with the modulation of cell cycle progression, which would allow cells to repair DNA damage, consequently resulting in apoptotic death [5,6]. Our studies have demonstrated that the DNA-damaging effect of UVB on HaCaT cells results in G1 phase arrest (Figure 4a,b). The exposure of HaCaT cells to UVB for 24 h resulted in the arrest of 58.43% of cells in the G1 phase, compared with 52.48% of cells in the control; however, CHS treatment reversed the UVB irradiation-induced decrease in the G1-phase cell population (Figure 4a). But CHS treatment did not show any significant difference for the S-phase cell population (Figure 4b). In the HaCaT cells, UVB exposure after 24 h resulted in a 29.28% G2/M-phase cell population compared, with 33.63% in the control, and was increased to the range of 42.16% to 48.59% (*p* < 0.001) with various concentrations of CHS. The CHS treatment groups had a higher proportion of cells in the G2/M phase compared to the control and UVB irradiation group (Figure 4b). CHS treatment directly affects the DNA repair process and regulates the cell cycle, thereby contributing to the promotion of cell proliferation.

### 3.4. CHS Reverses UVB Irradiation-Induced Cellular Senescence and Apoptosis

We next evaluated the protective effects of CHS in attenuating UVB irradiation-induced cell senescence via SA-β-Gal staining. The UVB group had more senescent cells than the control group, indicating that UVB irradiation induces cellular senescence (Figure 4c,d). Few senescent cells could be detected with CHS treatment in the HaCaT cells, demonstrating that CHS treatment decreased the number of senescent cells after UVB radiation (Figure 4c,d). Remarkably, CHS treatment led to a complete reversal of cell senescence, and exhibited a superior protective effect compared to the control group (Figure 4c,d). Moreover, AO/EB staining was used to identify cell apoptosis. Necrotic cells increased in volume and showed uneven red fluorescence at their periphery. We found that necrotic cells were dramatically increased following UVB irradiation compared to the control group (Figure 4e). The apoptotic rate of HaCaT cells in the CHS treatment group was decreased compared to that of the group without CHS treatment after UVB exposure (Figure 4f). And the increase in the apoptotic cell number was reversed after CHS treatment, with a dose-dependent improvement from 2.5% to 10% (Figure 4e,f). These results suggest that CHS treatment significantly decreased the cell death of UVB-irradiated cells through the inhibition of cellular senescence and apoptosis.

### 3.5. CHS-Induced Autophagy Activation after UVB Exposure

**Figure 5:**
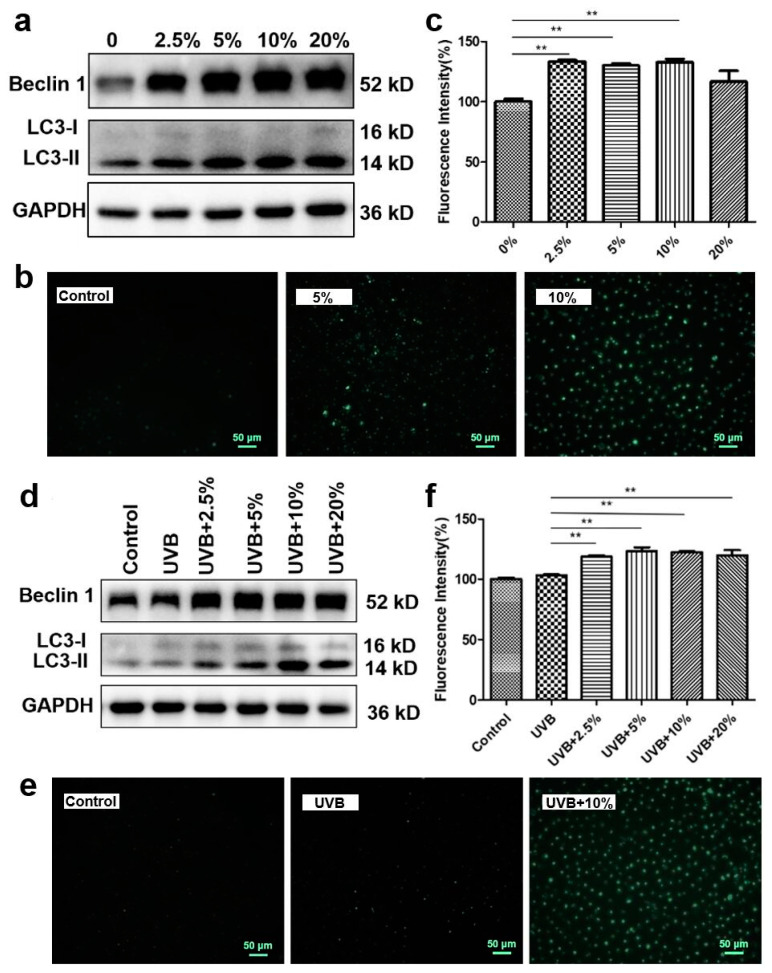
CHS induces cell autophagy in HaCaT cells following UVB exposure. (**a**) The protein expression levels of Beclin 1 and LC3 in HaCaT cells treated with CHS. (**b**) MDC staining images of HaCaT cells treated with CHS. (**c**) Fluorescence intensity of HaCaT cells treated with CHS. (**d**) The protein expression levels of Beclin 1 and LC3 in HaCaT cells exposed to UVB radiation with CHS treatment. (**e**) MDC-staining images of HaCaT cells treated with CHS. (**f**) Fluorescence intensity of HaCaT cells exposed to UVB radiation with CHS treatment. Scale bar, 50 μm. Results presented as means ± SD of three independent experiments (*n* = 3). ** *p* < 0.01.

Autophagy is a stress defense mechanism which maintains the balance of cells through the degradation of damaged cells by forming autophagosomes [19]. We investigated the effects of CHS on the formation of autophagosomes, as well as the expression of the autophagy-related factors Beclin-1 and LC3, in the HaCaT cells. Compared to the control group, treatment with 5%, 10%, and 20% CHS significantly enhanced the protein expression level of Beclin 1 and LC3-II (Figure 5a). The MDC staining results showed an increase in fluorescence intensity in the cells treated with CHS at concentrations of 2.5%, 5%, and 10% (*p* < 0.01, Figure 5b,c). Then, we examined the protein expression levels of Beclin 1 and LC3 in the HaCaT cells at 24 h after CHS treatment. Treatment with CHS upregulates cellular autophagy-related protein expression levels after UVB exposure (Figure 5d). A higher positive cell rate and more autophagosomes were observed in the CHS treatment groups after UVB exposure (*p* < 0.01, Figure 5e,f), which indicated that CHS treatment increased the level of autophagy. These results suggest that CHS treatment repairs UVB irradiation-induced cell damage through autophagy activation.

### 3.6. CHS Protects UVB Irradiation-Induced Skin Damage and Inflammatory Response

**Figure 6:**
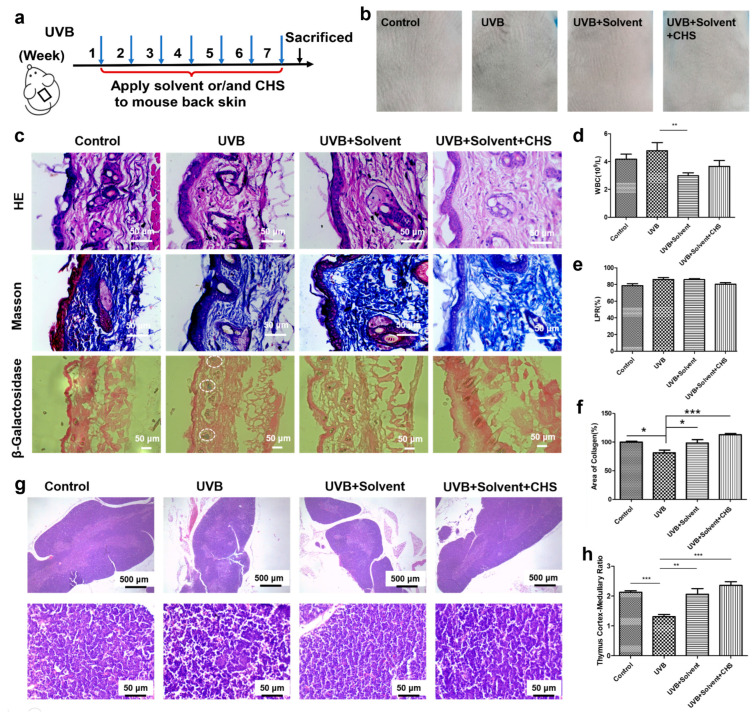
CHS repairs skin damage and protects thymus following UVB exposure in vivo. (**a**) The design of animal research. (**b**) Images of the mouse dorsal skin exposed to UVB radiation with CHS treatment. (**c**) HE, Masson’s trichrome, and β-Galactosidase staining images of skin tissue. (**d**) Density of white blood cells (WBCs). (**e**) Percentages of Langerhans cell precursors (LPRs) of hemocytes. (**f**) Relative area of collagen of skin tissue. (**g**) HE staining images of thymus (scale bar, 500 μm). (**h**) Thymus cortex/medullary ratio of thymus. Scale bar, 50 μm. Results presented as means ± SD of six independent experiments (*n* = 6). * *p* < 0.05, ** *p* < 0.01, *** *p* < 0.001.

The protective effect of CHS against UVB irradiation was observed in HaCaT cells. We next established a long-term UVB exposure for seven weeks to induce skin injury in the mouse skin model. A solvent with CHS was applied to repair skin injury in mice for six weeks after one week of UVB exposure (Figure 6a). Macroscopic images showed that the UVB-irradiated dorsal skin was considerably drier and paler than that of the control, whereas the morphological changes were markedly improved with CHS application (Figure 6b). CHS treatment rescued the increased scales observed with UVB irradiation-induced skin damage (Figure 6b). A histological analysis using HE staining showed that the UVB irradiation-induced increase in skin thickness, epidermal hyperplasia with enlarged sebaceous glands, and hyperkeratosis in mice could be rescued with the application of CHS (Figure 6c). Collagen, abundant in the skin dermis, can be measured using Masson’s trichrome staining. A reduction in collagen loss, fragmentation, and breakage was observed in the UVB-irradiated mice with CHS application (Figure 6c). In addition, β-Galactosidase staining showed a significant decrease in the number of senescent cells with CHS application after UVB exposure (Figure 6c). This suggests that topical treatment with CHS exhibited protective and therapeutic effects on reversing UVB irradiation-induced skin damage.

To test whether CHS could immediately protect against UVB irradiation-induced inflammatory responses, we measured the number of white blood cells (WBCs) and Langerhans cell precursors (LPRs). In all groups, WBC and LPR in normal range, indicated that treated with CHS on skin was safety (Figure 6d,e). Notably, we analyzed the morphology of the thymus, a primary lymphoid organ and the initial site for the development of immunological functions [20]. The thymus of the UVB-irradiated mice was significantly atrophied; the boundaries of the cortex and medulla were less clear than in normal mice, while the application of CHS could improve the pathological changes to the thymus (Figure 6f). The cortico/medullary ratio was decreased significantly after UVB exposure compared to the control group (Figure 6g). But groups treated with solvent, with or without CHS, had a higher cortico/medullary ratio than the UVB-irradiated group (Figure 5h). Taken together, these results suggest that the CHS had a positive effect on the improvement of the immune system.

## 4. Discussion

UVB irradiation is particularly harmful to human skin, leading to redness and a reduction in the skin’s elastic properties, and contributes to photodamage [21]. Repairing direct UVB irradiation damage is relevant to a number of skin conditions, including aging or photo-sensitive atopic dermatitis [22,23]. The application of stem cell factors exhibited a healing effect on UVB irradiation-damaged cells [24,25]. HUCMSCs are the most frequently used form of stem cell secretome, serving as a source of diverse growth factors associated with skin regeneration, such as growth differentiation factor 11 (GDF11), cell growth-stimulating factor (C-GSF), and collagen [26]. GDF11 contributes to the proliferation of human dermal fibroblasts (HDFs) and the improvement of skin structure [27], and C-GSF delivered from human amniotic MSCs exerts anti-inflammatory, pro-migrative, and proliferative effects on skin tissue [28]. To clarify the effects of the growth factors of HUCMSCs, we investigated the protective effects of a supernatant of HUCMSCs (abbreviated as CHS) on UVB-induced photodamage. The cell viability exhibited an increasing trend with different concentrations of the CHS treatment (less than 10%), indicating that CHS promotes cell growth in a dose-dependent manner. After exposure to 100 mJ/cm^2^ of UVB irradiation, 10% CHS was found to be the most efficient at restoring cell viability and enhancing cell migration. Keratinocyte migration is a critical step in wound healing, suggesting the regenerative efficacy of CHS against UVB irradiation in HaCaT cells. 

In the present study, a substantial increase in the number of senescent cells was observed after UVB irradiation, while treatment with CHS significantly reduced the number of senescent cells. Cellular senescence, which is characterized by an irreversible cell cycle arrest, might cause functional problems [29]. Consistent with our results, UVB irradiation-induced cellular senescence results from the slippage of long-term G2-arrested cells into the G1 phase [30]. We demonstrated the potential of CHS in regenerative medicine and highlighted the importance of the G2/M phase in preventing the transmission of DNA damage. DNA damage normally limits cellular proliferation through a temporary cell cycle arrest that facilitates the initiation of repair mechanisms to prevent the propagation of mutagenic lesions [31]. This indicates that CHS contributes to DNA repair and prevents the propagation of damaged DNA molecules. The stem cell secretome contains various biomolecules that contribute to tissue repair, such as exosomes [32]. Notably, it has been reported that MSC-secreted exosomes from culture media accelerate skin cell proliferation, migration, and the cell cycle [33]. Whether CHS exerts its protective effects on DNA damage through exosomes needs further investigation.

Previous studies have revealed that supernatants of MSCs restore the senescence of HDFs by enhancing cell migration, which is attributed to autophagy inhibition [34]. Autophagy, a conserved mechanism for maintaining intracellular homeostasis, is involved in several physiological processes, including the formation and maturation of the phagophore, autophagosome, and autolysosome [35,36]. Typically, autophagy can be activated by LC3 and Beclin1, both of which are biomarkers for autophagy. After exposure to UVB irradiation, CHS upregulated the autophagy protein expression level of LC3-II and Beclin 1 and increased the number of autophagosomes in the HaCaT cells, which may have contributed to the clearance of UVB-damaged organelles or proteins to maintain intracellular homeostasis. Moreover, autophagy is closely associated with apoptosis, which is crucial for regulating stem cell-dependent regeneration at the early stages of wound healing [37]. We also found that CHS reversed alterations in UVB irradiation-induced skin damage through apoptosis suppression. Other studies found that hucMSC-Ex treatment significantly increased HaCaT cell proliferation and migration in a time- and dose-dependent manner, and suppressed HaCaT apoptosis induced by H_2_O_2_ by inhibiting the nuclear translocation of apoptosis-inducing factor (AIF) [32]. These findings indicate that CHS has a repairing effect on UVB-induced damage via protective autophagy and the inhibition of apoptosis. 

UVB irradiation damages the integrity of the dermis by enhancing collagenase activity or disrupting procollagen biosynthesis, which leads to fragmented, disorganized collagen and a loss of collagen content [38]. Collagen is the major structural component of skin; its production or degradation could have profound effects on cutaneous functional integrity [21]. Studies have shown that supernatants of MSCs have vital roles in collagen production and skin tissue regeneration [39]. Long-term UVB exposure alters collagen in papillary dermis degradation; thus, we constructed a mouse model to investigate the effects of CHS on the dorsal skin of UVB-irradiated mice. We observed that mice treated with CHS exhibited significant improvements in wrinkle reduction, increased collagen content, and a reduction in senescent cells. Consistent with our result, several studies have found that MSC supernatants can safely and effectively promote the repair of light skin injury [40,41]. This may be associated with a higher content of collagen, transforming growth factor β (TGF-β), and vascular endothelial growth factor (VEGF), as well as its super cytokine transcriptional regulation ability [40]. BMSC-derived exosomes accelerate wound closure, re-epithelization, and collagen deposition through the regulation of the NF-E2-related factor 2 (NRF2) signaling pathway, associated with anti-inflammatory responses [41]. Multilineage-differentiating stress-enduring cells, isolated from human bone marrow-derived MSCs (BMSCs), not only exerted anti-inflammatory effects on lipopolysaccharide (LPS)-induced human HaCat cells, but also promoted wound healing and keratinocyte proliferation [42]. The protective effects of CHS may be associated with the suppression of inflammatory responses induced by UVB exposure. Moreover, ionizing radiation leads to different effects at the level of cells and organisms through inflammatory and immune response activation [43]. MSCs possess the capability to migrate to sites of tissue injury, thereby promoting wound healing through the secretion of paracrine factors that help to regulate immunity [44]. The thymus plays crucial roles in immunological function, and pathological changes in the thymus can significantly impact the immune system, resulting in immune dysregulation [20]. We further explored how CHS improves the pathological changes in the thymus after UVB exposure, suggesting that CHS has potential for tissue repair and regeneration, with improvements in immune function. 

In summary, our findings demonstrate that CHS exerts reparative effects on UVB exposure by promoting cell viability, facilitating cell migration, reducing senescent and apoptotic cells, and activating autophagy. Additionally, CHS application reduces wrinkles and senescent cells, increases collagen expression, and improves immune function in UVB exposure-induced skin photodamage. These results suggest that CHS has the potential to be developed as a novel therapeutic agent for UVB-induced skin photodamage. However, the molecular mechanisms of the protective effects of CHS are still unclear. Further investigations are essential to elucidate the underlying molecular mechanisms and optimize the dosage and administration time of CHS for maximum efficacy.

## 5. Conclusions

This study obtained the secretome from human umbilical cord mesenchymal stem cells (HUCMSCs), and investigated the reparative potential of CHS. We found CHS exhibited protective effects against UVB-induced damage by enhancing cell viability, mitigating senescence and apoptosis, and promoting DNA repair. Autophagy emerged as a key mechanism in CHS-mediated protection, evidenced by increased autophagy proteins post-UVB exposure. Moreover, CHS showed reparative effects on collagen integrity, reducing wrinkles and increasing the collagen content in UVB-irradiated mouse skin. This study extended its impact to immune function, revealing CHS’s potential in improving thymus pathology induced by UVB exposure, indicating its role in immune system regulation. Taken together, these findings underscore CHS as a promising therapeutic candidate for UVB-induced skin photodamage.

## Data Availability

The data presented in this study are available on request from the corresponding author.
